# Simulation-based education in neurology: lessons from the COVID-19 pandemic

**DOI:** 10.1186/s13104-023-06605-7

**Published:** 2023-11-02

**Authors:** Jaime Toro, Juan Sebastián Rivera, Jairo Gaitán, Daniela Rodríguez, Laura Andrea Serna-Corredor, Fabián Cortés-Muñoz, Thomas Medina, Manuel Yepes

**Affiliations:** 1 Department of Neurology, Hospital Universitario−Fundación Santa Fe de, Carrera 7 No. 117−15, Bogotá, Colombia; 2 School of Medicine, Universidad El Bosque, Carrera 7B Bis No. 132-11, Bogotá, Colombia; 3 School of Medicine, Universidad de Los Andes, Carrera 1 No. 18A−12, Bogotá, Colombia; 4 Multiple Sclerosis and other Neurological Disorders Research Group, Fundación Santa Fe de Bogotá, Carrera 7 No. 117-15, Bogotá, Colombia; 5 Fundación Clínica Shaio, Bogotá, Colombia; 6 Department of Neurology, Emory University School of Medicine, Atlanta, GA 30322 USA; 7 Department of Neurology, Veterans Affairs Medical Center, Atlanta, GA USA

**Keywords:** Medical education, Neurology, Simulated hospital, COVID-19 pandemic

## Abstract

**Objectives:**

The COVID-19 pandemic has led to the disruption of all sectors of the economy including education. According to UNESCO over 1.37 million young people including medical students, were affected by the lockdowns in response to COVID-19 and the subsequent closure of the education system. The primary challenge for medical education was to provide clerkships in a biosafety environment. This study aimed to determine the impact of a simulated hospital in a neurology clerkship of 5-year medical students during the coronavirus pandemic and compare their results with a non-pandemic group in Bogotá, Colombia.

**Results:**

The students in the pandemic group answered a Likert scale survey regarding their satisfaction with the simulated hospital. Both groups were required to perform an oral, mid-term and final examination. From the results, it is clear that students perceived that exposure to a simulated hospital facilitated their learning process (93.1%) and allowed greater interaction with the teacher compared to a face-to-face environment (77.3%). There were no clinically significant differences in test results. This experience indicates that a simulated hospital is a valuable method to acquire clinical skills in trainees, that could be integrated into the curricular milestones of medical education programs regardless of the pandemic.

**Supplementary Information:**

The online version contains supplementary material available at 10.1186/s13104-023-06605-7.

## Introduction

COVID-19 the etiologic agent of SARS-COV2 (Severe Acute Respiratory Syndrome-Coronavirus type 2) was declared as a pandemic disease by the WHO (World Health Organization) on March 11th, 2020 [1]. As of November 2021 there were more than 253 million confirmed cases of COVID-19 [1]. Colombia was the fourth country in the region with the highest incidence per 100,000 inhabitants [2, 3]. According to the United Nations Educational, Scientific and Cultural Organization (UNESCO), over 138 countries completely closed their educational systems representing around 1.37 million students [4].

In response to COVID-19, medical education has been seeking alternative teaching methods focused on virtual lectures and online simulation practice. Despite these initiatives, students are at a disadvantage when it comes to acquiring practical clinical skills [5–7]. Notwithstanding, efforts and research have been focused on postgraduate students compared to undergraduate trainees [8–12].

This study aims to describe the response to the emerging educational challenges implemented by the school of medicine of Universidad de los Andes (Bogotá, Colombia). A simulated hospital (SH) was developed with educational tools, including trained actors who could replicate symptoms and signs, allowing students to learn clinical skills such as anamnesis and physical examination. This hospital applies the principles that simulation could reduce the probability of error and improve the training of medical practitioners in a safe and controlled environment that replicates a real-world experience [13–17]. The findings of this study may constitute a good starting point for discussion and potential implementation of this educational model, even beyond this overwhelming scenario.

## Main text

### Methods

#### Study design and sample

A quasi-experimental design was conducted on a population of 5th-year medical students during their neurology clerkship at the Universidad de los Andes. Our sample comprised two similar groups across a different time period. Participants in the first group, which will be referred to as the non-pandemic group throughout the article, were composed of 57 students who received a traditional face-to-face format during the second semester of 2019. The second group, which will be referred to as the pandemic group throughout the article, was composed of 46 students who received a mixed virtual and simulation-based clerkship in the second semester of 2020.

The courses were essentially identical except for the delivery method. To reduce heterogeneity between both groups, theoretical and practical components were included and the same supplementary resources were provided. In addition, classes were taught by the same attendants and the entire clerkship lasted the same number of weeks.

#### Neurology clerkship

The neurology clerkship for both groups included synchronous and asynchronous learning. The non-pandemic group’s synchronous learning component was a neurology clinical rotation. Students were exposed to neurology ward patients, daily rounds, seminars and journal clubs. On the other hand, the pandemic group’s synchronous learning component included SH activities, where professional actors recreated pathological situations. Additionally, students in the pandemic group were required to attend different virtual activities based on the neurology competencies addressed in the non-pandemic group’s face-to-face format.

The asynchronous component encompasses the learning resources provided at the beginning of the course to both groups. Participants had access to textbooks, articles and neurological exam videos suggested by the attendants.

#### Simulated hospital

A SH recreated different environments that resembled emergency rooms and inpatient or outpatient care settings. During this clerkship, professional actors depicted several neurological disorders using a realistic screenplay developed by experts. Before each instance, the actors met with a neurologist who validated the standardized patient’s characteristics and provided feedback before, during and after the performance.

The students were able to interview the standardized patient and perform a clinical history and neurological examination. All of the details of the anamnesis and neurologic exam were done in a controlled environment, observed and guided by an attendant. After a thorough debriefing, the attendant provided objective feedback. The assessment strategy for the student performance was the ability to give a reliable history, perform a complete neurologic examination, localize lesions and elaborate on a differential diagnosis and treatment. Finally, empathy, communication abilities and interpersonal skills were also explored.

#### Measurement of the acquired knowledge

To evaluate theoretical knowledge acquisition, students in the pandemic and the non-pandemic groups were required to take a mid-term and a final examination through a virtual platform. To minimize heterogeneity between both sets of data, all the assessments comprised questions of the same complexity. Additionally, an oral exam was conducted to measure the practical skills of both cohorts. Each exam was measured using a scale from 0.0 to 5.0.

#### Satisfaction evaluation

Students in the pandemic group answered two 11-question Likert scale surveys regarding their satisfaction with the virtual clerkship and the SH adapted from a routine satisfaction survey undertaken by the Universidad de los Andes. This assessment explored learning abilities and other features such as effective feedback, difficulties treating actors as real patients, interaction with teachers and the overall quality of the information received. Likewise a similar 10-question Likert-type scale survey was given to the neurologists who attended the SH. The questionnaire evaluated the satisfaction of teachers regarding their perception of the SH and its effectiveness as a teaching tool. Equivalent questions about the learning environment, interaction with the students and the ability of the actors to represent a real patient were asked.

### Statistical analysis

To check the normality of the distribution of quantitative variables, a Shapiro–Wilk test was employed. Central tendency statistics (means) and dispersion (standard deviation) were employed to describe normal variables, otherwise median and interquartile ranges were used. Due to the non-normal distribution of continuous variables and to compare medians between pandemic and non-pandemic groups, a Wilcoxon rank sum test was performed. In the case of qualitative dichotomous variables, the difference in proportions Z-test was used.

Furthermore, scores on oral, mid-term and final exams were modeled using a classical linear regression. Two models were constructed for the analysis. The first model was a bivariate model that included the subject’s group (pandemic or non-pandemic) as the only independent variable. The second model was a multivariate model that included the covariates sex (Female = 1, Male = 0), and age (Year) to evaluate the incremental value of academic performance based on exposure levels. A bidirectional stepwise (forward and backward elimination) technique was used to select the covariates. Both models were evaluated through the coefficient of determination and the significance of each was evaluated using a student t-test. A p-value ≤ 0.05 was considered statistically significant and all analyses were performed using Stata/SE 17.1 analytical software (StataCorp, College Station, TX).

## Results

Sociodemographic characteristics of subjects in both groups are shown in the first section of Table 1. The pandemic group was statistically younger than the non-pandemic participants (− 1.49 years apart, p < 0.0001). Both groups were similar with respect to sex.

**Table 1:** Comparison of sociodemographic characteristics and test scores by exams between study groups

**Sociodemographic Characteristics**				
Characteristics	Group		p-Value	
	Pandemic (n = 46)	Non-pandemic (n = 57)		
Age (years)				
Median (IQR)	23 (22–24)	24.49 (23.40–25.30)	< 0.0001*	
Sex (n -%)				
Female	22–47.83	34–59.65	0.2311**	
**Comparison of Test Scores by Exam Type and Study Group.**				
Type of Exam	Group		p-Value	
	Pandemic (n = 46)	Non-pandemic (n = 57)		
Oral Exam				
Median (IQR)	4 (3.8–4.3)	4.3 (4.2–4.5)	0.0002*	
Final Exam				
Median (IQR)	3.3 (3–3.4 )	3.2 (2.6–3.6)	0.2438*	
Mid-Term Exam				
Mean (SD)	2.77 (0.54)	3.07 (0.66)	0.0167***	

Abbreviations:

IQR: interquartile range

SD: standard deviation

*Difference calculated using the Wilcoxon Rank-Sum Test

**Difference calculated using Z-test for proportions

***Differences calculated using Z-test for difference of means

The satisfaction of both groups regarding the teaching method was one of our main outcomes. For this purpose, we employed survey data concerning virtual clerkship satisfaction. According to our results, most of the students had good internet access and an electronic device at home. In addition, most of them considered the seminars and clinical cases valuable for their learning process. However, our results demonstrate that the main obstacle faced by the students was their ability to stay focused on their virtual classes, which affected their emotional state of mind and utmost performance. Virtual clerkship Satisfaction Likert Scale results are shown in Table 2.

**Table 2:** Virtual Clerkship Satisfaction Likert Scale results (n = 46)

**Virtual Clerkship Satisfaction Likert Scale results (n= 46)**					
Questions	Strongly disagree	Disagree	Neutral	Agree	Strongly agree
I have good quality internet access available when I need it.	1 (2.17%)	2 (4.34%)	7 (15.21%)	26 (56.52%)	10 (21.73%)
It is easy for me to focus on virtual classes.	1 (2.17%)	12 (26.08%)	12 (26.08%)	15 (32.6%)	6 (13.04%)
It is possible to have an interaction with the teachers, and that allows me to ask questions in virtual activities.	0	2 (4.34%)	5 (10.86%)	24 (52.17%)	15 (32.6%)
The technological and audiovisual tools used during the virtual classes worked adequately.	0	1 (2.17%)	6 (13.04%)	26 (56.52%)	13 (28.26%)
I perceived that most of the teachers who taught me were comfortable with virtuality.	0	3 (6.52%)	6 (13.04%)	21 (46.65%)	16 (34.78%)
Emotionally, I feel good about the virtual teaching model.	0	10 (21.7%)	14 (30.43%)	17 (36.95%)	5 (10.86%)
The virtual seminars improved my skills as a general practitioner in the area of neurology.	0	1 (2.17%)	3 (6.52%)	29 (63.04%)	13 (28.26%)
The journal club fostered my interest in the topic seen.	0	3 (6.52%)	4 (4.34%)	31 (67.39%)	8 (17.39%)
The clinical cases fostered my interest in the topic seen.	0	0	1 (2.17%)	35 (76.08%)	10 (21.73%)
The essay topics were relevant to my medical training.	0	1 (2.17%)	4 (4.34%)	31 (67.39%)	10 (21.73%)
**Simulated hospital satisfaction Likert Scale for students and teachers**					
**Students Satisfaction Survey (n = 44)**					
Questions	Strongly disagree	Disagree	Neutral	Agree	Strongly Agree
The interaction with the simulated patients was satisfactory and adequately recreated a real clinical scenario.	0	2 (4.5%)	11 (25%)	20 (45.5%)	11 (25%)
Feedback after simulation activities was adequate.	0	0	4 (9.1%)	21 (47.7%)	19 (43.2%)
The simulated hospital allowed me to develop clinical decision skills that are applicable to a real clinical scenario.	0	0	6 (13.6%)	20 (45.5%)	18 (40.9%)
I believe that the simulated hospital tool should be incorporated into the medicine program regardless of the pandemic.	0	1 (2.3%)	4 (9.1%)	16 (36.4%)	23 (52.3%)
I think that the simulated hospital facilitated my learning.	0	0	3 (6.8%)	24 (54.5%)	17(38.6%)
I consider that the simulated hospital allows me to have a greater interaction with the teachers, compared to a face-to-face environment	0	3 (6.8%)	7 (15.9%)	16 (36.4%)	18 (40.9%)
**Teachers Satisfaction Survey (n = 7)**					
Questions	Strongly disagree	Disagree	Neutral	Agree	Strongly Agree
I consider that a simulation-based hospital is an effective tool for transmitting knowledge to students	0	0	0	3 (42.9%)	4 (57.1%)
I believe that the simulated hospital model should be incorporated into the medical school curriculum regardless of the pandemic.	0	2 (28.6%)	0	3 (42.8%)	2 (28.6%)
The simulated hospital benefits the students by developing neurological competencies/skills as a general practitioner.	0	0	0	6 (85.7%)	1 (14.3%)
I consider that the actors adequately represented the clinical characteristics required by the script	0	0	3 (42.9%)	2 (28.6%)	2 (28.6%)
I consider the time spent with students under this simulation-based education model to be adequate.	0	0	1 (14.3%)	4 (57.1%)	2 (28.6%)

Most of the students considered that the SH is a useful addition that should be incorporated into their curriculum regardless of the pandemic. Students considered debriefings provided adequate feedback and increased teacher-student interaction (Table 2). A similar pattern of results was obtained in the questionnaire of the neurology attendants (n = 7). All of them agreed that this is an effective tool for transmitting knowledge to students as well as developing neurology skills needed as general practitioners (Table 2). Additionally, they felt comfortable teaching and evaluating the students on the oral exam. Despite the general satisfaction with the SH, a similar conclusion was reached by the neurologists and students regarding the actors’ ability to perform the clinical characteristics required by the script.

Finally, statistically significant differences were found in the oral and midterm exam scores between the groups (Table 1). The results were higher in the non-pandemic group (+ 0.3 points in the oral (p = 0.0002) and midterm exam (p = 0.0167)) compared to the pandemic group. Non-statistically significant differences were observed in the results of the final exam.

Bivariate and multivariate models are consistent with the results reported in Table 1. The results showed that changing from the non-pandemic to pandemic group represented a decrease of -0.468 points in the oral exam (p < 0.001) and − 0.53 points in the mid-term exam (p = 0.028) after adjusting by confounding variables (Table 3). Despite being statistically significant, these results are not particularly different in a real-world situation, making the scores of both groups comparable. In all the cases, the models demonstrated they were a good fit for the data.

**Table 3:** Bivariate and multivariate linear regression models by exam type

Bivariate models									
Variable	Oral Exam (R2 = 0.113) = 4.58			Mid-Term Exam (R2 = 0.05) = 3.28			Final Exam (R2 = 0.01) = 2.91		
	k	95% CI	*P value*	k	95% CI	*P value*	k	95% CI	*P value*
Group	-0.282	-0.43, -0.12	*< 0.001*	-0.29	-0.53, -0.05	*0.017*	*0.133*	*-0.09, 0.36*	*0.257*
Multivariate models									
	(R2 = 0.150 ) = 5.25			(R2 = 0.05 ) = 3.28			(R2 = 0.017) = 2.76		
*Group*	-0.308	-0.468, -0.148	**< 0.001**	-0.285	-0.539, -0.031	**0.028**	0.147	-0.096, 0.391	0.234
*Age*	-0.027	− 0.058, 0.003	0.084	0.0009	− 0.048, 0.049	0.969	0.003	-0.043, 0.050	0.882
Sex	0.056	-0.102, 0.214	0.485	0.080	− 0.0171, 0.331	*0.529*	*0.080*	*-0.161, 0.321*	*0.512*

95% CI = 95% confidence interval

Bolded values represent statistical significance at a 95% confidence level

## Discussion

During the COVID-19 pandemic the main challenge for medical education was to offer high-quality clerkships within a biosafe environment. As a solution to this challenge, most countries worldwide opted for virtual education models [2, 5, 18–20]. Electronic-learning research has shown numerous positive effects in education, such as the availability of a vast array of resources and accessibility from a variety of locations without compromising students’ security, making it suitable for the pandemic scenario. However, with this model undergraduate and postgraduate medical students identified a variety of concerns regarding their learning process, including technological challenges, diminished emotional well-being, decreased interaction between students and instructors and most importantly, difficulty with the practice of clinical skills [7, 19, 21–23].

The SH emerges as an alternative that allows the exposure of students to clinical cases within a biosafety environment. It also provides neurology trainees with an alternative to improve their clinical skills when hospital access is difficult. Participants felt that the SH allowed the development of skills for clinical decision-making, communication abilities, neuroanatomical localization and the development of competencies as general practitioners.

The emotional wellbeing of students was a major concern in the literature regarding virtual education during the pandemic and was thus a secondary outcome to be measured in this study. Our results showed that 52% of the students did not feel emotionally well with a virtual-only learning modality, a result consistent with what has been reported [7]. The SH might help with a number of issues affecting the emotional state of students, such as reducing the distractions inherent to virtual education, enhancing the learning experience and promoting peer interaction.

Another advantage of the SH is that students perceived that this experience permitted better interaction with their instructors. In this environment other frequent actors in clinical practice, such as medical residents, did not divert the attention of teachers allowing direct interaction between the student, neurology attendants and the standardized patient. Along these lines, teachers can solve students’ questions and provide formative feedback, one of the pillars of education that strengthens the knowledge acquired [24].

This experience ensures exposure to neurologic diseases despite the difficulties and limitations of not being in a face-to-face environment. Our clerkship demonstrates that simulation-based practice in neurology, with the addition of virtual tools, can improve medical knowledge and develop confidence in the management of a variety of neurological pathologies needed as general practitioners. This model is not only an alternative for pandemic education but also an excellent teaching complement for medical curricula. A similar conclusion is supported by the fact that we did not find a clinically significant difference between the performance in the different exams that were conducted to evaluate theoretical knowledge. Our results indicate that the SH appears to have an influence on the students’ acquired knowledge and may be an excellent complement to conventional medical education.

## Conclusions

The present study shows that a SH is a valuable method to acquire clinical skills in trainees with improvement in medical knowledge, satisfaction and comparable results in academic evaluations. This experience suggests that exposure to a SH could be integrated into medical education program’s milestones regardless of the pandemic.

### Limitations

Due to the quasi-experimental nature of this study, the absence of randomization was one of its most significant limitations. However, we attempted to control this by adjusting for confounding variables. Because of the unpredictability of the pandemic’s onset, each group could be exposed to unknown circumstances that may influence the effect of intervention. Another limitation of this study is related to the validation of the surveys used to assess satisfaction, which were not specifically designed for our study. Future studies may consider validating the survey instrument for use in similar contexts.

### Electronic supplementary material

Below is the link to the electronic supplementary material.

Supplementary Material 1: Virtual clerkship survey (Spanish and English)

Supplementary Material 2: Simulated hospital survey for teachers (English)

Supplementary Material 3: Simulated hospital survey for students (English)

Supplementary Material 4: Simulated hospital survey for students (Spanish)

Supplementary Material 5: Simulated hospital survey for teachers (Spanish)

## Data Availability

The datasets generated and/or analysed during the current study are available in PAPYRUS, the repository associated to Universidad de los Andes, http://papyrus-datos.co/dataset.xhtml?persistentId=doi:10.57924/YQKBE2.

## List of Abbreviations

COVID-19: Coronavirus disease 2019
UNESCO: United Nations Educational, Scientific and Cultural Organization
SARS-COV2: Severe Acute Respiratory Syndrome-Coronavirus type 2
WHO: World Health Organization
SH: Simulated Hospital
